# Cypin Inhibition as a Therapeutic Approach to Treat Spinal Cord Injury–Induced Mechanical Pain

**DOI:** 10.1523/ENEURO.0451-23.2024

**Published:** 2024-02-14

**Authors:** Nisha K. Singh, Srinivasa R. Gandu, Lun Li, Li Ni, Cigdem Acioglu, Ersilia Mirabelli, Liam L. Hiester, Stella Elkabes, Bonnie L. Firestein

**Affiliations:** ^1^Department of Cell Biology and Neuroscience, Rutgers, The State University of New Jersey, Piscataway, New Jersey 08854; ^2^Molecular Biosciences Graduate Program, Rutgers, The State University of New Jersey, Piscataway, New Jersey 08854; ^3^Department of Neurosurgery, New Jersey Medical School, Rutgers, The State University of New Jersey, Newark, New Jersey 07101

**Keywords:** cypin, guanine deaminase, neuropathic pain, pharmacological inhibition, spinal cord injury, von Frey test

## Abstract

Cypin (cytosolic postsynaptic density protein 95 interactor) is the primary guanine deaminase in the central nervous system (CNS), promoting the metabolism of guanine to xanthine, an important reaction in the purine salvage pathway. Activation of the purine salvage pathway leads to the production of uric acid (UA). UA has paradoxical effects, specifically in the context of CNS injury as it confers neuroprotection, but it also promotes pain. Since neuropathic pain is a comorbidity associated with spinal cord injury (SCI), we postulated that small molecule cypin inhibitor B9 treatment could attenuate SCI-induced neuropathic pain, potentially by interfering with UA production. However, we also considered that this treatment could hinder the neuroprotective effects of UA and, in doing so, exacerbate SCI outcomes. To address our hypothesis, we induced a moderate midthoracic contusion SCI in female mice and assessed whether transient intrathecal administration of B9, starting at 1 d postinjury (dpi) until 7 dpi, attenuates mechanical pain in hindlimbs at 3 weeks pi. We also evaluated the effects of B9 on the spontaneous recovery of locomotor function. We found that B9 alleviates mechanical pain but does not affect locomotor function. Importantly, B9 does not exacerbate lesion volume at the epicenter. In accordance with these findings, B9 does not aggravate glutamate-induced excitotoxic death of SC neurons in vitro. Moreover, SCI-induced increased astrocyte reactivity at the glial scar is not altered by B9 treatment. Our data suggest that B9 treatment reduces mechanical pain without exerting major detrimental effects following SCI.

## Significance Statement

Neuropathic pain is a debilitating comorbidity associated with spinal cord injury (SCI). Available pharmacological therapies are ineffective or have adverse effects. The development of new and targeted drugs that can effectively alleviate neuropathic pain is urgently needed. Cypin is the primary guanine deaminase in the central nervous system and an essential enzyme in the purine salvage pathway. Activation of the purine salvage pathway leads to the production of uric acid, which promotes pain but also neuroprotection. We found that inhibition of cypin post-SCI alleviates mechanical pain. The inhibitor does not prevent the spontaneous recovery of locomotor function or exacerbate lesion volume and astrogliosis at the injury epicenter. Thus, cypin inhibitors could be promising therapies for the treatment of neuropathic pain.

## Introduction

Individuals with spinal cord injury (SCI) experience loss of motor, sensory, and autonomic functions and neuropathic pain, which decrease quality of life (van Leeuwen et al., 2012; Pacheco Barzallo et al., 2020). SCI involves a primary phase that results in physical damage to the spinal cord (SC) and a secondary phase during which pathological molecular and cellular alterations exacerbate damage caused by the initial trauma. Excitotoxic neuronal death is among the essential mechanisms contributing to secondary injury at the lesion site. The manifestation of SCI-induced neuropathic pain below the injury level is partly the consequence of increased neuronal excitability, maladaptive synaptic plasticity, loss of inhibitory processes, and changes in glial activation in the dorsal horn of SC in regions remote from the epicenter (Shiao and Lee-Kubli, 2018). Although SC stimulation, neuromodulation, and activity-based interventions are promising approaches for the alleviation (Bandres et al., 2022; Chen et al., 2022; Dorrian et al., 2023) or prevention (Chhaya et al., 2019) of neuropathic pain, currently available pharmacological treatments do not effectively attenuate neuropathic pain or have severe side effects. Therefore, the development of new therapeutics is urgently needed.

Cypin [cytosolic postsynaptic density protein 95 interactor; guanine deaminase (GDA)] plays a critical role in the purine salvage pathway, which mediates the production of uric acid (UA). UA is an antioxidant with protective effects in CNS injury and neuronal injury models in vitro (Scott et al., 2005; Du et al., 2007; Yu et al., 2009; Swiatkowski et al., 2018; Singh et al., 2021). Paradoxically, increased UA levels are implicated in pain (Kelley et al., 1967; Martinon et al., 2006; Fang et al., 2013). Therefore, we postulated that inhibitors of the GDA activity of cypin attenuate SCI-induced pain sensitivity, potentially by preventing excess UA buildup in the injured SC. Moreover, guanosine, a guanine-based purine (GBP) produced upstream of this pathway (Yuan et al., 1999; Paletzki, 2002; Akum et al., 2004; Fernandez et al., 2008), promotes antinociception after SCI (Schmidt et al., 2008, 2009, 2010a,b; de Oliveira et al., 2016). Thus, cypin inhibition may contribute to the alleviation of pain after SCI by the accumulation of upstream GBPs and decreased UA levels in the SC.

Here, we examine the pain-attenuating effects of a cypin inhibitor after SCI. Since activation of cypin confers protection against glutamate-induced excitotoxicity in cultured hippocampal and cortical neurons, we investigated whether inhibition of cypin exacerbates excitotoxicity-induced SC neuronal loss in vitro or negatively affects astrocyte reactivity at the glial scar. We report that inhibition of the GDA activity of cypin after SCI alleviates mechanical pain sensitivity in the hindlimbs without affecting motor function. Treatment with the inhibitor does not alter lesion volume or injury-induced increase in astrocyte reactivity at the epicenter. Moreover, the inhibitor does not exacerbate excitotoxicity-evoked SC neuronal loss in vitro. Thus, we report, for the first time, that inhibition of guanine metabolism at the level of cypin is a viable strategy for decreasing mechanical neuropathic pain after SCI. Our results also support the notion that the inhibitor does not exert major adverse effects that negatively impact injury outcomes.

## Materials and Methods

### Ethical approval for animal studies

The work was approved by and performed in accordance with guidelines of the Rutgers University Institutional Animal Use and Care Committee (PROTO999900080 and PROTO999900765) and adheres to the Guide in the Care and Use of Laboratory Animals established by the US National Academy of Sciences. Female mice were used for all studies.

### SC contusion injury

Contusion injury was induced as described previously (David et al., 2014). Briefly, 8-week-old female C57BL/6 mice were anesthetized with ketamine (100 mg/kg; Vedco) and xylazine (10 mg/kg; Akorn) via an intraperitoneal (i.p.) injection and bupivacaine (2 mg/kg) via a subcutaneous injection. A laminectomy was performed at the T8 level, and a moderate contusion injury (60 kdyne) was induced using the Infinite Horizon impactor device (Precision Systems and Instrumentation). Sham-injured mice were anesthetized and laminectomized at T8. Subcutaneous injections of Lactated Ringer’s solution (1 ml; Baxter International), Baytril (0.25 mg/kg; Bayer), and buprenorphine SR (1 mg/kg) were administered to all mice immediately after surgery. Buprenorphine SR was administered to the mice once every 3 d for 7 d, and all other postoperative treatments were administered once daily for 7 d. In addition, the expression of bladders for injured mice was performed manually twice each day for the entire duration of the experiment. Following surgery and/or contusion injury, the Basso mouse scale (BMS) was used to evaluate open-field locomotor activity 1, 2, 7, 14, and 21 dpi (Basso et al., 2006). Pain evaluation by von Frey filament test was performed at 21 dpi.

### Structure and preparation of drugs

**Figure 1.**
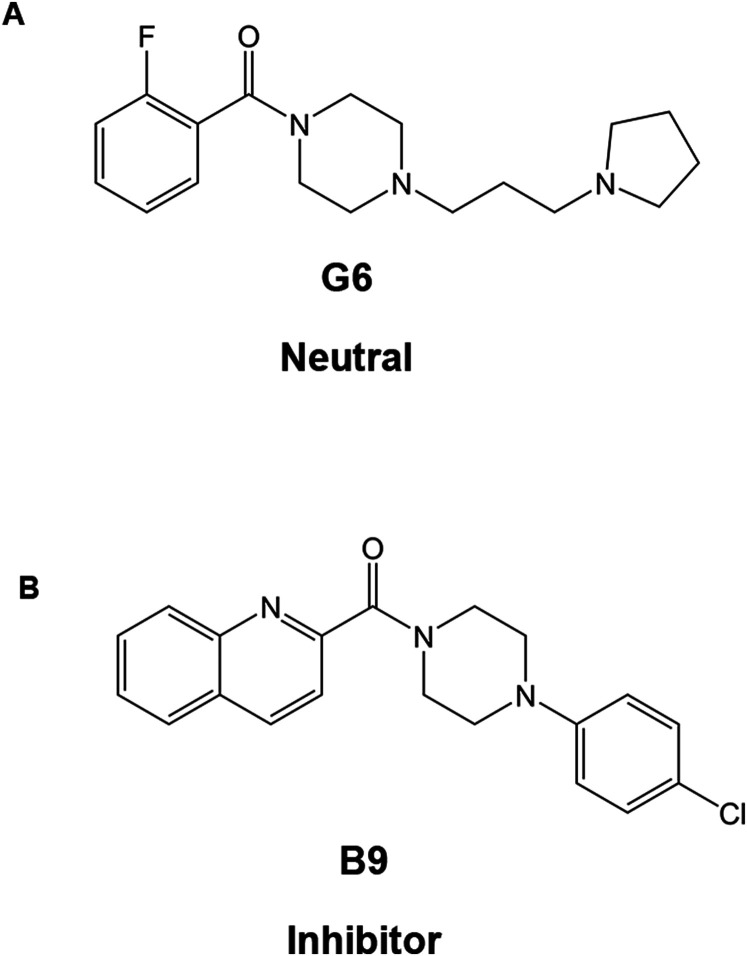
Structures of the small molecule compounds used in this study. ***A***, Structure of G6, a compound that does not affect the initial rate of the GDA reaction promoted by cypin activity. ***B***, Structure of B9, a compound that decreases the initial rate of the GDA reaction promoted by cypin activity. The identification of compounds is discussed in detail by Swiatkowski et al. (2018).

Stock solutions of B9 (cypin inhibitor; Fig. 1), H9 (cypin activator; Fig. 1), and G6 (neutral compound; Fig. 1) were prepared in 100% DMSO and stored at −80°C as previously described (Swiatkowski et al., 2018).

### Intrathecal injection of B9

Intrathecal delivery of B9 and vehicle occurred at 1, 3, and 7 dpi. Mice were anesthetized with isoflurane, and a 27-gauge needle was inserted into the interlaminar space between L5 and L6 for injection (6 µl). Drugs were administered at a concentration of 0.5 mg/kg in 100% DMSO.

### von Frey filament test

Testing was performed on an elevated wire mesh platform in a plexiglass observation chamber. Mice were habituated to the observation chamber in 1 h daily intervals for 2 d leading up to testing. Immediately before the test, mice were placed into the chamber for 30 min for acclimatization. To assess mechanical sensitivity, right and left hindpaw withdrawals upon probing with a set of von Frey filaments (ranging from 0.008 to 2.0 g; North Coast Medical) were evaluated using the up–down paradigm described previously (Khariv et al., 2017; Mirabelli et al., 2019).

### Immunohistochemistry and quantitation of SC tissue

Mice were subjected to transcardial perfusion with saline and 4% paraformaldehyde (PFA) in phosphate-buffered saline (PBS) at 21  dpi. Spinal cords were postfixed in PFA in PBS overnight, cryoprotected in 27% sucrose in PBS, embedded in optimal cutting temperature compound, and flash-frozen in dry ice (David et al., 2014). Tissue from the lumbar dorsal horn (LDH) was then sectioned transversely at a thickness of 30 µm. Fixed sections were blocked in 10% normal goat serum, 10% Triton X-100, and 2% sodium azide in PBS. Tissue was then incubated for 1 h at room temperature with primary antibodies for glial fibrillary acidic protein (GFAP; 1:5,000; Dako, catalog #GA524; RRID: AB_2811722) or ionized calcium-binding adaptor molecule 1 (Iba1; 1:1,000; Millipore, catalog #MABN92; RRID: AB_10917271). The tissue was imaged using a Nikon A1R confocal microscope. For labeling of cypin-positive neurons, we immunostained the sections using rabbit anticypin (Swiatkowski et al., 2018) and chicken antimicrotubule-associated protein 2 (MAP2; Novus Biologicals, catalog #NB300-213; RRID: AB_2138178). Complementary fluorescent secondary antibodies were used for visualization, and nuclei were stained with Hoechst 33342 dye (Thermo Fisher Scientific). NIH ImageJ software was used for quantification of the mean fluorescent intensity of each marker in a defined region of interest (ROI) within each tissue section in the LDH. All data were normalized to the ROI area and control group.

### Analysis of lesion volume

As described above, injured mice were subjected to transcardial perfusion, and dissected spinal cords were subjected to overnight postfixation, cryoprotection, embedding, and flash freezing (David et al., 2014). Tissue from the SCI epicenter was then sectioned transversely at a thickness of 30 µm. Fixed sections were blocked and then incubated with a GFAP primary antibody (1:5,000; Dako, catalog #GA524; RRID: AB_2811722) to label astrocytes at the glial scar (lesion border). A complementary fluorescent secondary antibody was used for visualization, and nuclei were stained with Hoechst 33342 dye (Thermo Fisher Scientific). The tissue was imaged using the EVOS FL microscope. NIH ImageJ software was used for the quantification of the lesion area and total SC section area for every third tissue section spanning the entire length of the lesion. Lesion volume and SC volume (for the region of the lesion) were calculated, and the lesion volume for each animal was normalized to the respective SC volume.

### SC cell culture

SC cultures were grown as we previously described (Du et al., 2007; Ramadan et al., 2021). Cells were plated on poly-d-lysine (0.1 mg/ml; Sigma-Aldrich)–coated glass coverslips in 24-well plates at a density of 100,000 cells/well. SC cultures were grown in serum-containing medium (SCM; 90% Dulbecco's modified Eagle serum containing 10% heat-inactivated horse serum) for 6 d at 37°C and 5% CO_2_. The neurons in the culture are primarily sensory neurons (Zhang et al., 2009).

### Glutamate-induced excitotoxicity

On day in vitro 6, SC cultures were injured with 100 µM glutamate in SCM for 1 h. After injury, SCM containing glutamate was removed and replaced with recovery medium (1:1 conditioned:fresh SCM), and cultures were incubated at 37°C and 5% CO_2_ for 24 h prior to fixation. Cultures were treated with small molecules for 1 h after glutamate-induced injury for 24 h. Cultures were then fixed with 4% PFA in PBS for 15 min at room temperature and incubated with blocking solution (2% normal goat serum, 10% Triton X-100, 2% NaN3, in PBS) for 1 h at room temperature. To assess neuronal viability, we incubated cultures with polyclonal mouse anti-MAP2 primary antibody (1:500; Fisher Scientific, catalog #BDB556320) for 1 h, then with Alexa Fluor 647 goat anti-mouse secondary antibody (1:500) or Alexa Fluor 488 goat anti-mouse secondary antibody (1:500) for 1 h, and Hoechst 33342 dye (1:1,000) for 15 min.

Ten images were taken of each coverslip using the EVOS FL Cell Imaging System. Neurons immunolabeled for MAP2 were manually counted using NIH ImageJ analysis software, and the number of viable neurons per well was compared between conditions. All analyses were performed with the experimenter blinded to the condition. GraphPad Prism was used to perform a ROUT outlier test followed by repeated measures ANOVA or one-way ANOVA and Tukey's multiple-comparisons test.

### Protein analysis by Western blotting

Mice were killed by CO_2_ inhalation, decapitated, and the SC was dissected. A 5 mm segment containing the epicenter was excised. LDH tissue was also dissected. Epicenter and LDH tissue were homogenized using a motorized pestle in 50 μl ice-cold lysis buffer [10 mM HEPES buffer, 10% sucrose, and 5 mM ethylenediaminetetraacetic acid (EDTA) at pH 7.0] supplemented with PhosSTOP Phosphatase Inhibitor (Sigma-Aldrich) and cOmplete Mini EDTA-Free Protease Inhibitor Cocktail (Sigma-Aldrich) tablets. Tissue lysates were centrifuged at 800 × *g* for 5 min at 4°C to pellet cellular nuclei. The supernatant was collected and then centrifuged at 20,000 × *g* for 45 min to separate soluble proteins and membrane-bound proteins. The supernatant containing soluble proteins was stored at −80°C until use.

Sample protein concentrations were quantified using the Pierce BCA Protein Assay (Thermo Fisher Scientific). Equal amounts of lysate protein were run on SDS-PAGE and transferred to PVDF membranes. Total protein was visualized using the REVERT Total Protein Stain Kit (VWR) and the Li-Cor Odyssey Fc imaging system. The membranes were blocked in 5% bovine serum albumin (5% BSA) in TBST (25 mM Tris, 3 mM KCl, 140 μM NaCl, 0.05% Tween 20, pH 7.4) and probed using primary antibodies for the following proteins: GFAP (1:1,000; mouse; NeuroMab; catalog #75-240; RRID: AB_10672299) or cypin [1:1,000; rabbit; Firestein Laboratory (Swiatkowski et al., 2018)], Complementary horseradish peroxidase–conjugated secondary antibodies were used for visualization upon development with Immobilon Western Chemiluminescent HRP Substrate (Millipore). Protein bands were visualized using the Li-Cor Odyssey Fc imaging system and quantified with Image Studio Lite Version 5.2 software. Protein signals were normalized to total protein and then normalized to the control condition prior to analysis.

### Statistical analyses

All statistical analyses were performed using GraphPad Prism 9 software. Statistical differences were determined using Student's *t* test, one-way ANOVA or two-way ANOVA followed by Tukey's multiple-comparisons test, as indicated in figure legends. Prior to analysis, outliers were removed using the ROUT outliers test.

## Results

### Intrathecal treatment with the cypin inhibitor, B9, ameliorates mechanical pain without affecting the spontaneous recovery of locomotor function following SCI

**Figure 2.**
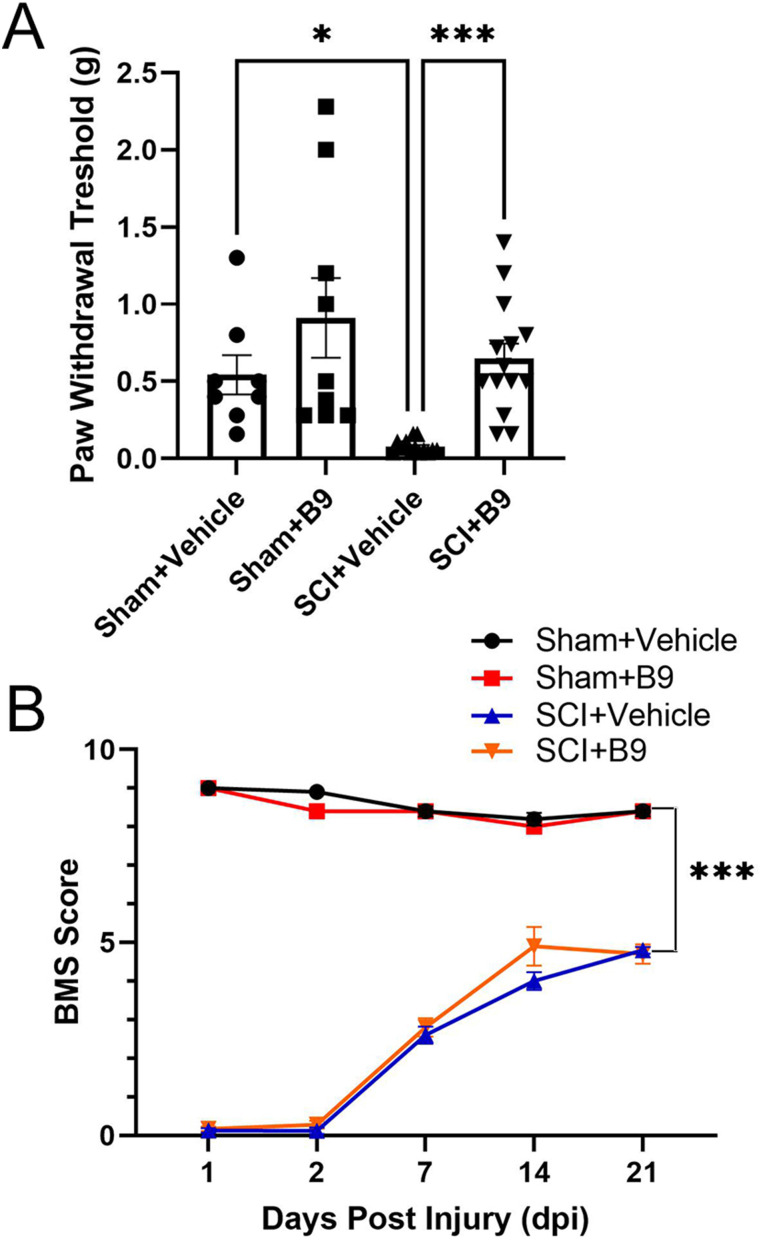
Treatment with cypin inhibitor, B9, attenuates SCI-induced mechanical pain sensitivity, but does not alter spontaneous recovery of locomotor function. ***A***, Paw withdrawal thresholds assessed by the von Frey filament test at 21 dpi. B9 treatment did not have significant effects on sham-injured mice, whereas it restored SCI-induced decrease in paw withdrawal thresholds to sham values. *n* = 8–12. *p < 0.05, ***p < 0.001 as determined by one-way ANOVA followed by Tukey's multiple-comparisons test. ***B***, Open-field locomotor function evaluated by the BMS score. Mice that sustained a SCI manifested spontaneous recovery of locomotor function over 21 days. The recovery was partial, and BMS scores remained significantly different than sham values. A representative experiment is shown. The experiment was repeated three times and yielded similar results. The values are presented as mean ± SEM. *n* = 5, ***p < 0.001 by ANOVA repeated measures followed by Tukey's multiple-comparisons test.

To determine whether inhibition of the GDA activity of cypin leads to changes in SCI-induced neuropathic pain, mice that sustained a moderate midthoracic contusion injury or sham injury were treated with vehicle or the cypin inhibitor, B9, administered intrathecally at 1, 3, and 7 dpi. Mechanical pain in the hindlimbs was assessed by the von Frey filament test at 21 dpi, the time when spontaneous recovery of locomotor function is in progress. SCI significantly decreased the paw withdrawal threshold in vehicle-treated mice compared with vehicle-treated sham controls, indicating increased mechanical pain sensitivity, whereas B9 treatment restored paw withdrawal thresholds to control (sham) values (Fig. 2*A*). B9 treatment did not have any effect on sham mice.

We also tested whether treatment with B9 has positive or negative effects on locomotor function. Open-field locomotor function was evaluated by assessing BMS scores starting at 1 dpi until 21 dpi. B9 did not alter locomotor function in sham controls. In mice sustaining an SCI, BMS scores were 0 on days 1 and 2 pi but showed progressive recovery on the subsequent days, reaching ∼5 by 21 dpi. We did not observe significant differences in spontaneous recovery of locomotor function between vehicle- or B9-treated injured mice (Fig. 2*B*). Thus, our data suggest that B9 treatment specifically targets mechanical pain pathways, but not motor pathways, after SCI.

**Figure 3.**
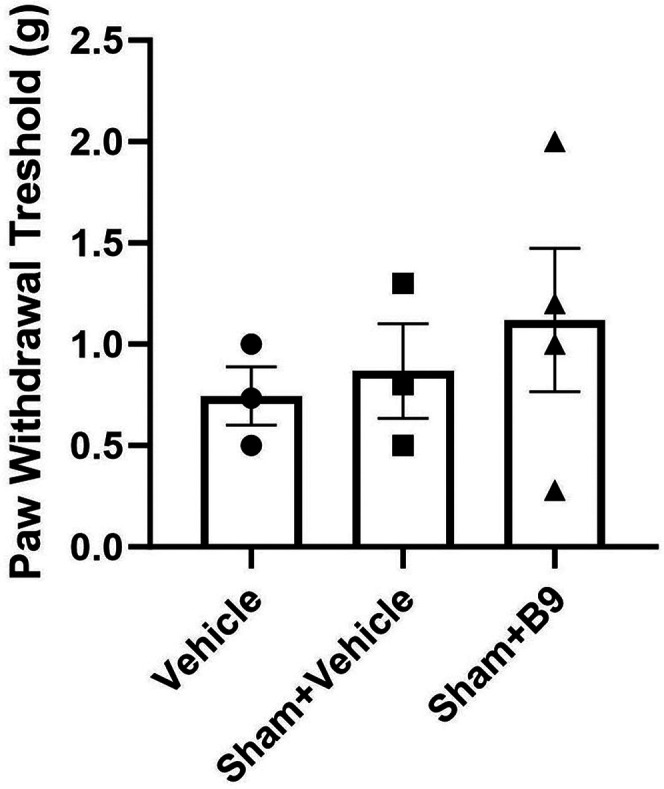
The response of naive, vehicle- or B9-treated sham mice to mechanical pain. Mechanical pain does not significantly differ between naive or vehicle-treated sham mice. Additionally, B9 treatment does not affect pain in sham mice. Therefore, in the rest of our studies, we did not include naive mice.

In our experiments, we did not include naive mice because the paw withdrawal thresholds of vehicle or B9-treated sham rats were not different than those of naive mice (Fig. 3).

### B9 treatment does not increase lesion volume or exacerbate glutamate-induced excitotoxicity in SC neuronal cultures

**Figure 4.**
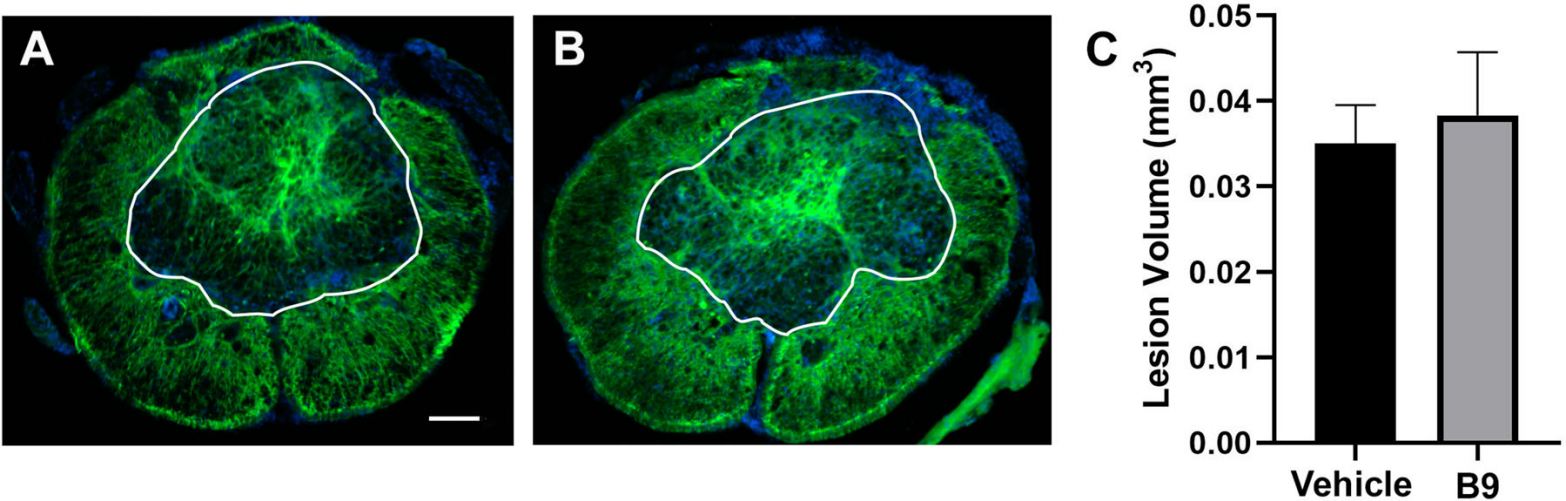
Treatment with B9 does not alter lesion volume following SCI. ***A***, Photomicrographs of representative transverse SC sections at the injury epicenter immunostained for GFAP to outline the lesion. The white line delineates the lesion. ***B***, Quantification of lesion volume. Data were analyzed by Student's *t* test. Outliers were removed prior to statistical analysis by the ROUT test. The values are presented as mean ± SEM. *n* = 3. Scale bar = 100 μm.

Because activation of cypin leads to neuroprotection in cultured hippocampal and cortical neurons (Tseng and Firestein, 2011; Swiatkowski et al., 2018), we postulated that inhibition of cypin can attenuate neuroprotection and, in doing so, exacerbate cell and tissue loss at the injury epicenter. This, in turn, could increase lesion volume. Therefore, we determined whether B9 treatment alters lesion volume at 21 dpi. Thoracic transverse SC sections that included the injury epicenter were immunostained for GFAP to demarcate the lesion border. The area devoid of GFAP immunoreactivity was considered to be the lesion core. No differences were detected in lesion volume between injured mice treated with B9 or vehicle (Fig. 4). Thus, B9 does not exacerbate tissue loss at the lesion site.

**Figure 5.**
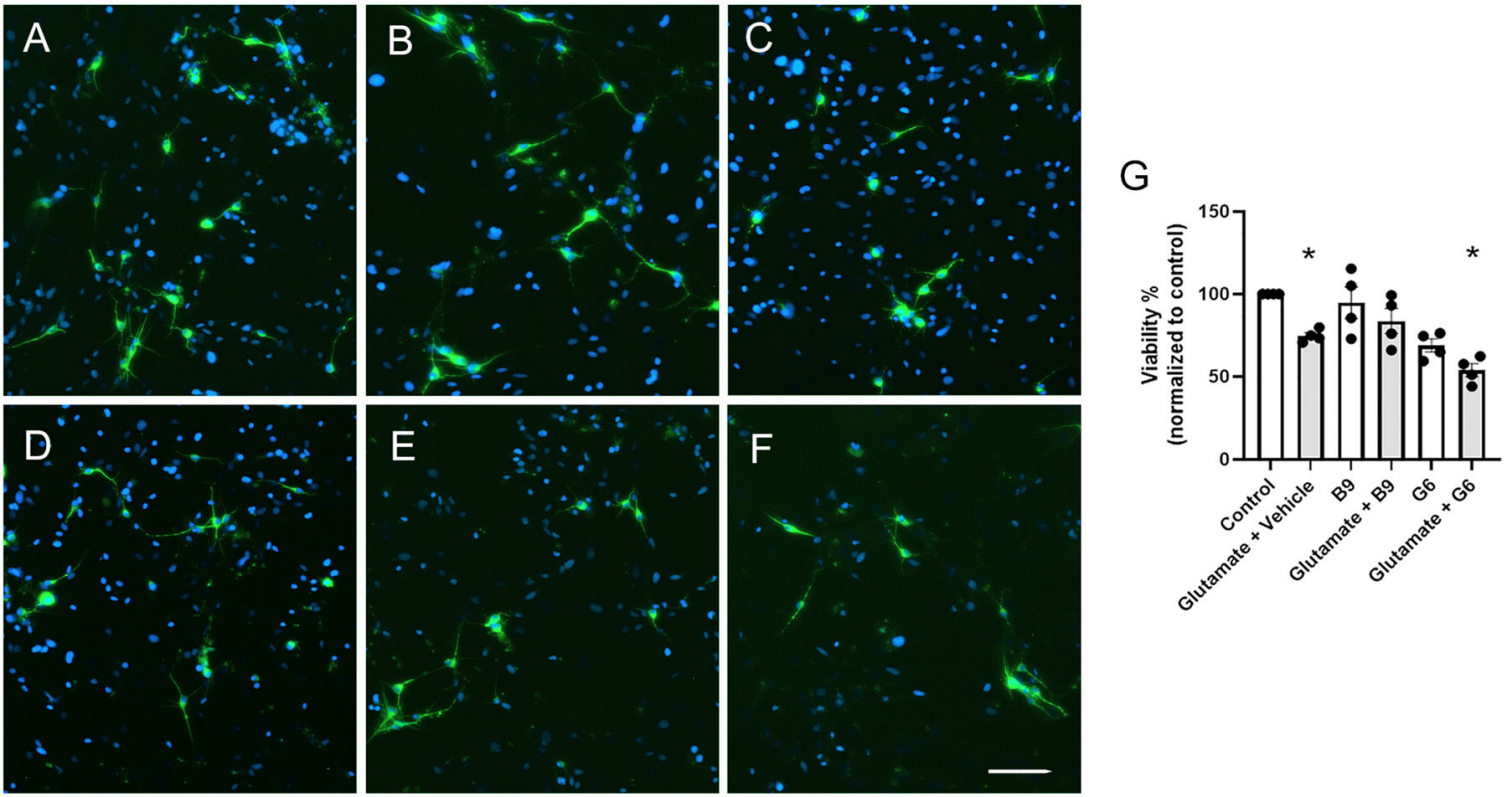
B9 does not exacerbate excitotoxic loss of SC neurons in vitro. SC neurons were treated with (***A***) vehicle (control), (***B***) B9, (***C***) G6, (***D***) glutamate, (***E***) glutamate + B9, and (***F***) glutamate + G6. Neurons were immunolabeled with antibodies against MAP2 (green). Hoechst 33342 staining (blue) was used to visualize cell nuclei. ***G***, The number of viable cells was quantified 24 h after treatment. The values are presented as mean ± SEM. *n* = 4. *p < 0.05 as determined by one-way ANOVA followed by Tukey's multiple-comparisons test.

Since glutamate-induced excitotoxicity has been implicated as a major cause of neuronal loss at the injury epicenter and cypin activation in brain neurons is neuroprotective (Tseng and Firestein, 2011; Swiatkowski et al., 2018), we postulated that inhibition of cypin could worsen excitotoxicity-induced loss of SC neurons in vitro. Treatment of neurons with glutamate significantly decreased neuronal viability; however, the addition of B9 did not exacerbate excitotoxicity-induced neuronal loss (Fig. 5). Taken together, our results indicate that B9 treatment does not exert major adverse effects on tissue integrity and neuron viability.

### B9 treatment does not alter astrocyte reactivity at the injury epicenter

**Figure 6.**
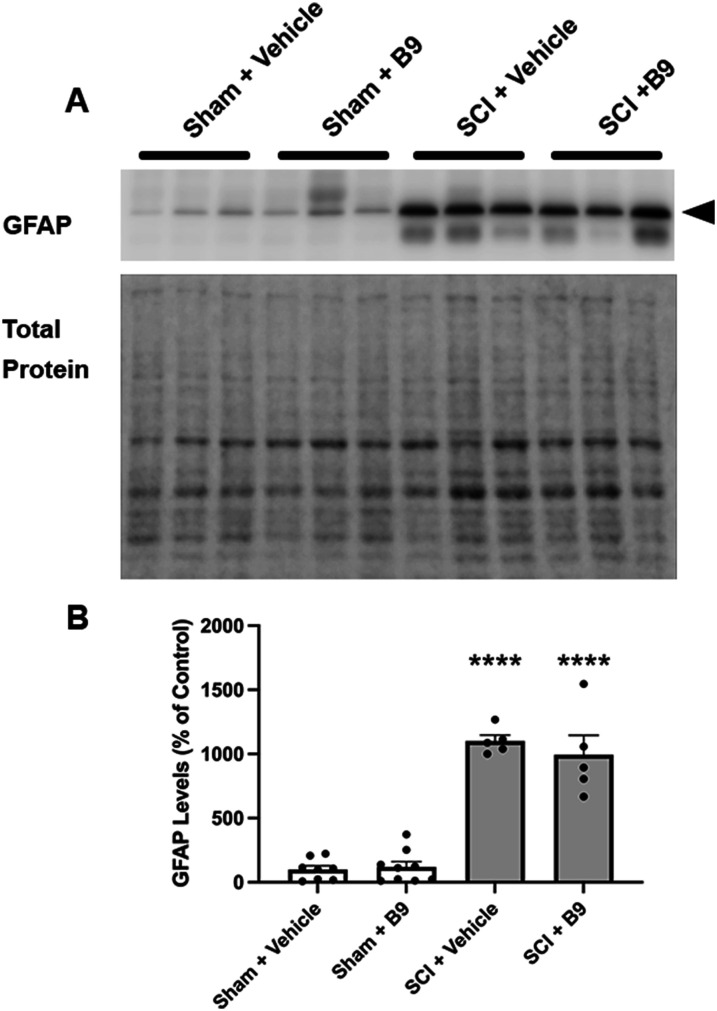
B9 treatment does not alter astrocyte reactivity at the lesion site. ***A***, Western blot showing a band at the molecular weight corresponding to GFAP (arrowhead) in vehicle- or B9-treated sham and injured mice (top panel). The bottom panel shows the total protein loaded on each lane, which was used for normalization. ***B***, Quantification of the bands corresponding to GFAP after normalization to total protein and presented as percent of control (sham + vehicle). GFAP levels were significantly increased in the injury epicenter following SCI but were not affected by B9 treatment. *n* = 5–8. ****p < 0.0001 significantly different from vehicle- and B9-treated mice as determined by ANOVA followed by Tukey's multiple-comparisons test.

Reactive astrocytes are the major cell type in the glial scar that forms at the injury epicenter. Both beneficial and detrimental roles have been attributed to the glial scar and reactive astrocytes (reviewed in Ding et al. (2021)). Among the detrimental roles is the impediment of axonal regrowth, partly due to the release of inhibitory factors by reactive astrocytes [reviewed in Costa et al. (2022)]. Astrocyte activation and astrogliosis are paralleled by increased GFAP expression. It is well-known that GFAP protein levels are upregulated at the injury epicenter following SCI (Sofroniew, 2014). We therefore investigated whether B9 treatment alters astrocyte reactivity and astrogliosis at the injury epicenter at 21 dpi by quantifying GFAP levels in SC segments that contain the injury epicenter. As expected, Western blot analysis demonstrated a significant increase in GFAP levels at the lesion site of injured mice treated with a vehicle. B9 treatment did not have any significant effects on injury-induced increase in GFAP levels, suggesting that astrocyte reactivity or astrogliosis at 21 dpi is not altered by B9 treatment (Fig. 6).

### B9 treatment does not alter glial reactivity in the LDH

**Figure 7.**
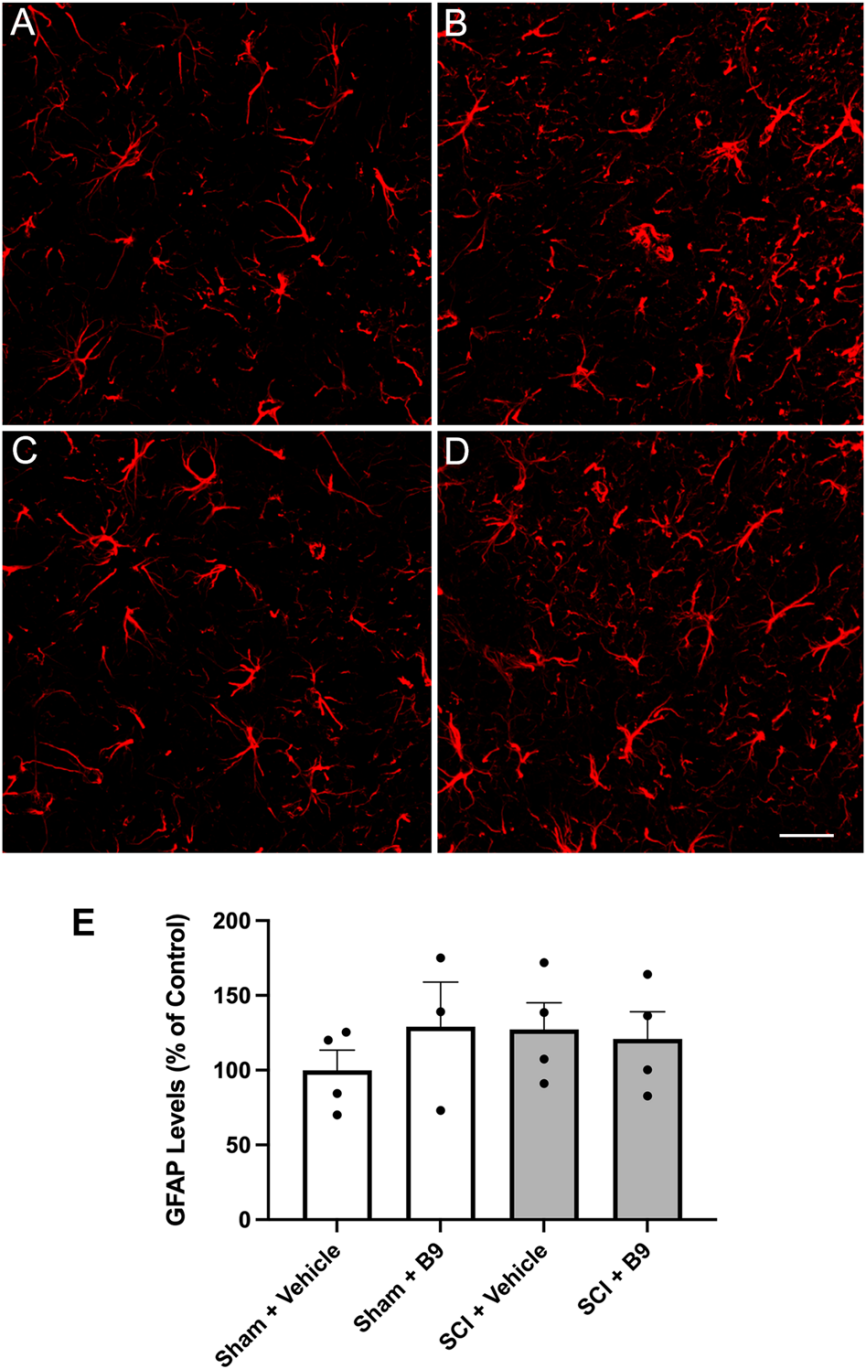
B9 treatment does not alter GFAP levels in the LDH. Representative images of transverse SC sections showing GFAP immunoreactive cells in the LDH (***A***) sham + vehicle, (***B***) sham + B9, (***C***) SCI + vehicle, and (***D***) SCI + B9. ***E***, Quantification of GFAP fluorescent signal. Four sections per mouse were analyzed and averaged. Data were then normalized to the sham + vehicle (control) group and analyzed by one-way ANOVA followed by Tukey's multiple-comparisons test. Outliers were first removed by the ROUT test. Data are presented as mean ± SEM. *n* = 3–4. Scale bar = 100 μm.

**Figure 8.**
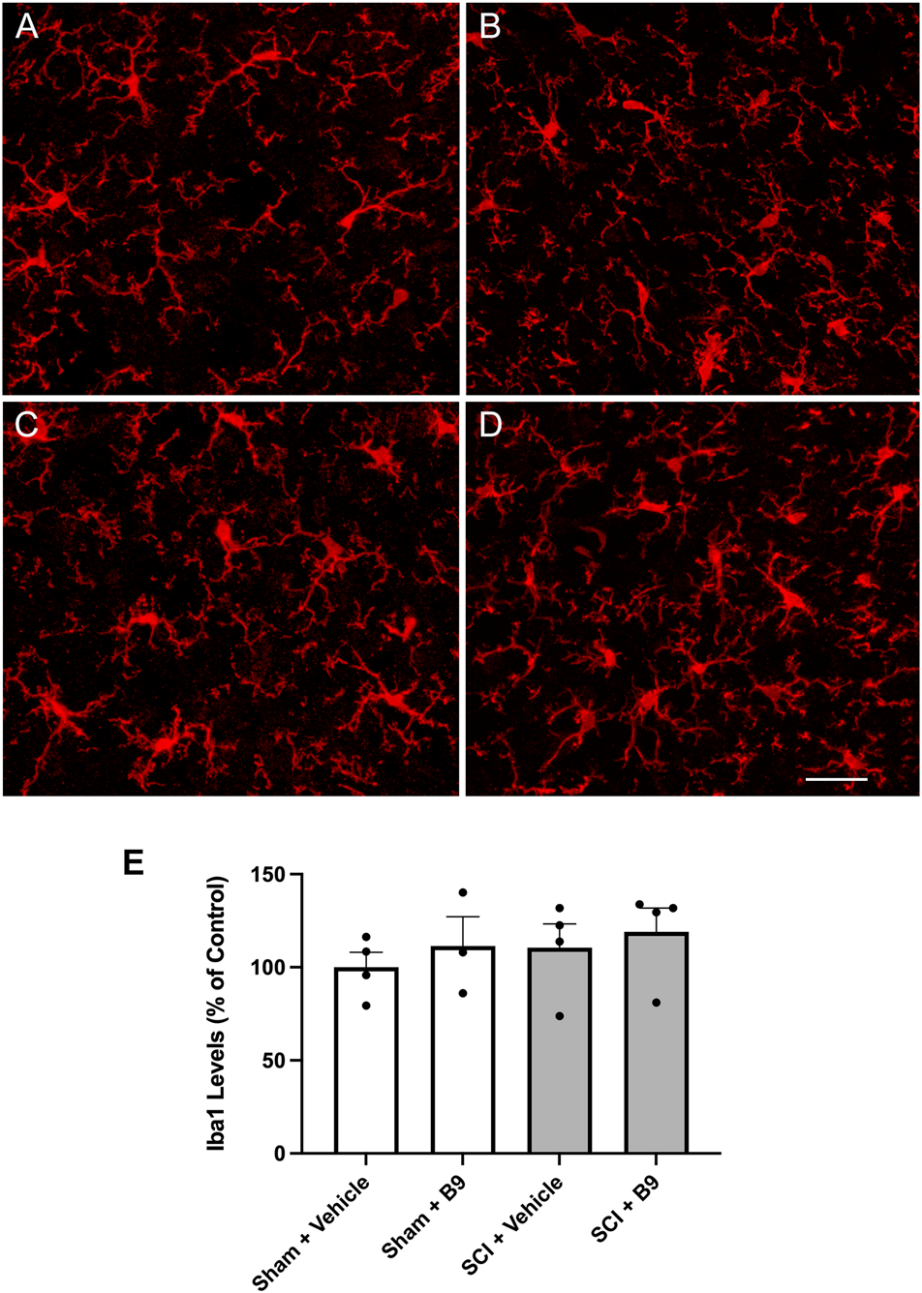
B9 treatment does not alter Iba-1 levels in the LDH. Representative images of transverse SC sections showing Iba-1 immunoreactive cells in the LDH (***A***) sham + vehicle, (***B***) sham + B9, (***C***) SCI + vehicle, and (***D***) SCI + B9. ***E***, Quantification of Iba1 fluorescent signal. Four sections per mouse were analyzed and averaged. Data were then normalized to the sham + vehicle (control) group and analyzed by one-way ANOVA followed by Tukey's multiple-comparisons test. Outliers were first removed by the ROUT test. Data are presented as mean ± SEM. *n* = 3–4. Scale bar = 100 μm.

We initiated studies to determine the mechanisms by which B9 ameliorates neuropathic pain. Activated microglia and reactive astrocytes have previously been implicated in neuropathic pain mechanisms (Shiao and Lee-Kubli, 2018). Since the LDH is the region that receives and integrates pain information from the hindlimbs, we investigated whether B9 reduces pain sensitivity by attenuating glial activation in the LDH following thoracic SCI. We immunolabeled LDH sections obtained from sham controls and injured mice treated with vehicle or B9 (Figs. 7, 8). Quantification of fluorescent signal in the LDH at 21 dpi showed no significant differences in GFAP or Iba-1 immunoreactivity between sham controls and mice sustaining SCI. This finding is in conceptual agreement with our earlier report showing that GFAP expression, which is significantly increased in the LDH at 8 dpi, returns to sham levels by 28 dpi (Pallottie et al., 2018). Treatment with B9 did not alter GFAP or Iba-1 immunoreactivity in the LDH of sham controls or injured mice. Thus, modulation of glial reactivity by B9 is not a mechanism underlying the beneficial effects of B9 on mechanical pain sensitivity. However, an effect at an earlier time point cannot be ruled out.

### B9 treatment does not alter total cypin protein levels in the epicenter or superficial layers of the LDH postinjury

**Figure 9.**
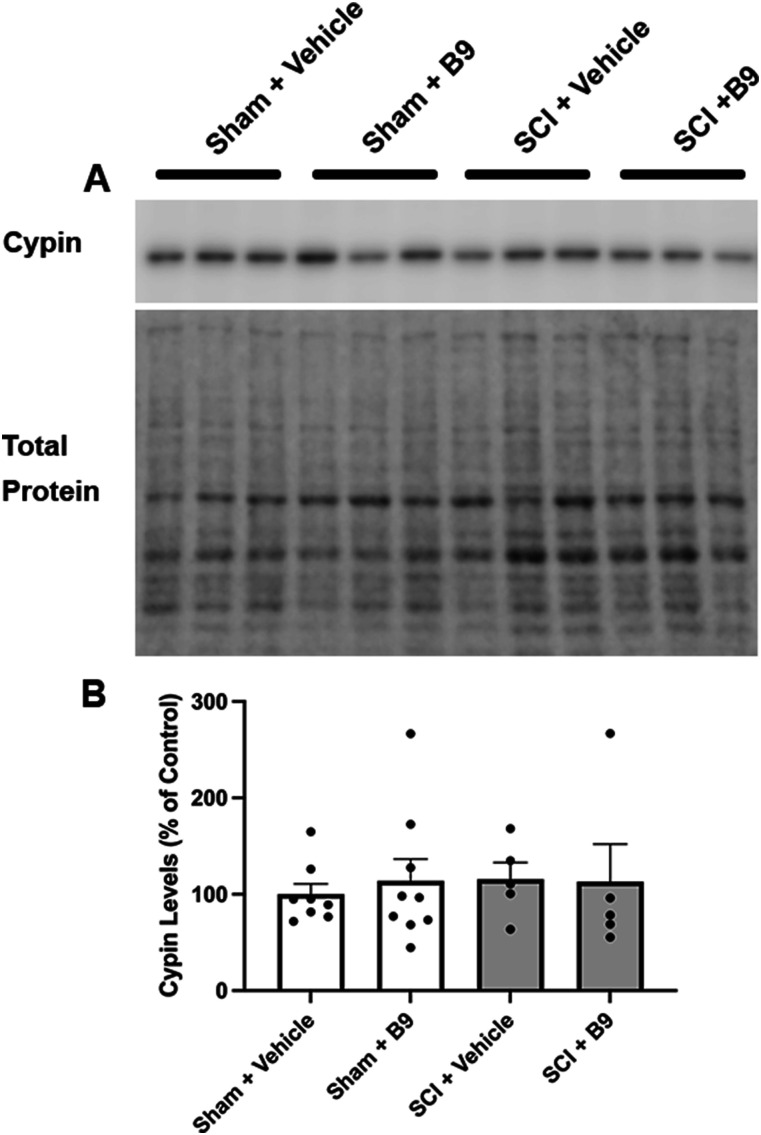
Cypin expression at the lesion site is not modulated by SCI or B9. ***A***, Representative Western blot showing a band at the molecular weight corresponding to cypin (top panel). Total protein was used for normalization (bottom panel). ***B***, Quantification of the bands in the Western blots after normalization to total protein and expressed as a percent of control (vehicle-treated sham). *n* = 5–9.

Modulation of cypin protein levels by SCI and B9 could be a mechanism that regulates the pain response since changes to cypin levels alter UA production, which in turn, affects neuroprotection and pain sensitivity. We therefore assessed cypin protein levels at the epicenter and LDH. Western blot analysis of tissue obtained from SC segments that include the epicenter showed that cypin levels are not altered by SCI or B9 treatment of injured mice (Fig. 9).

**Figure 10.**
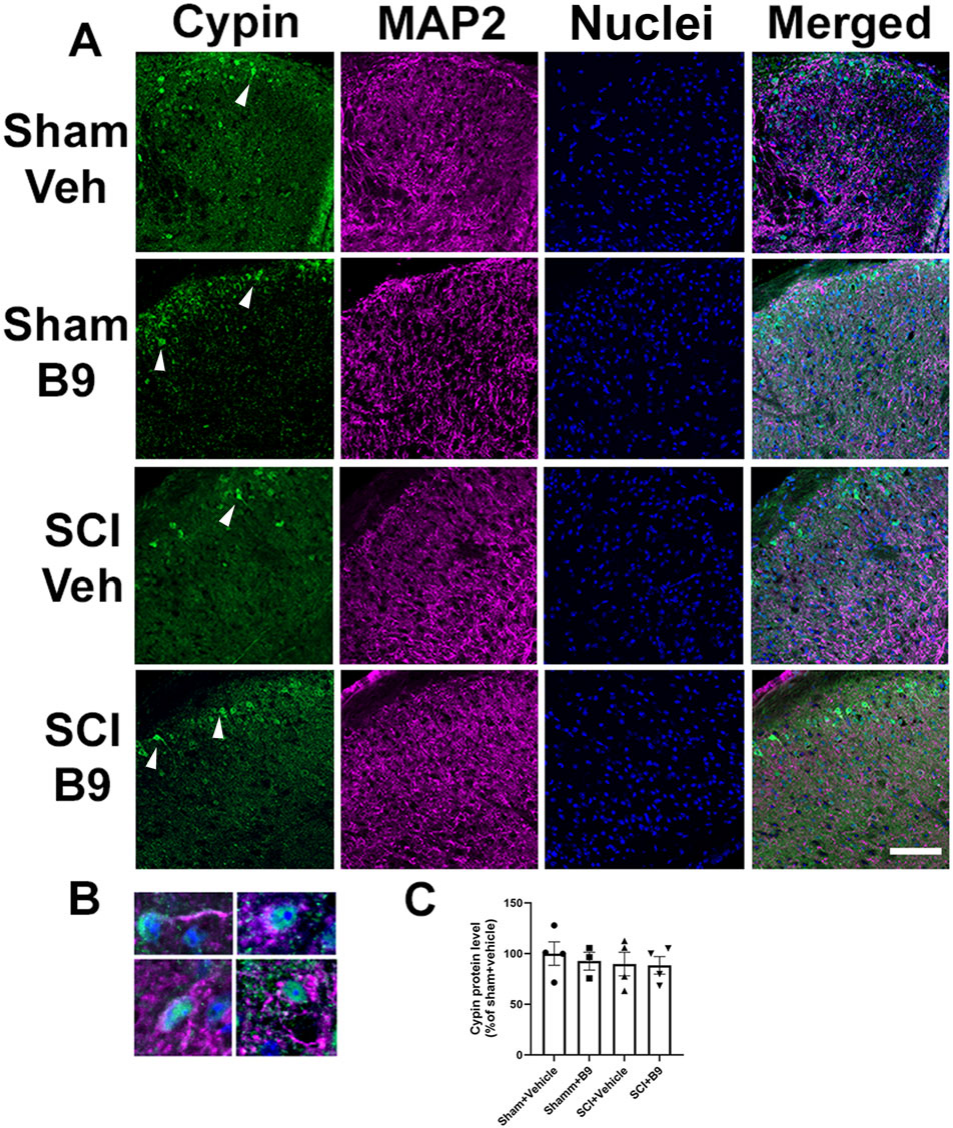
Cypin immunoreactivity localizes to SC neurons in the LDH superficial layers and cypin levels are not altered by SCI or B9 treatment. ***A***, Transverse SC sections were immunolabeled with antibodies against cypin (green) or MAP2 (magenta). Hoechst 33342 staining (blue) was used to visualize cell nuclei. Most cypin-positive cells are localized in the superficial layers of the SC (arrows). ***B***, High magnification picture of neurons expressing cypin. ***C***, Quantification of cypin immunoreactivity in the superficial layers of the LDH demonstrates that B9 treatment does not affect cypin expression. Scale bar = 100 µm.

To assess cypin levels in the LDH and to study the localization of cypin-positive neurons in the LDH, transverse lumbar SC sections were triple-labeled with antibodies against cypin and MAP2 (a neuronal marker found in processes) and Hoechst dye to visualize nuclei. Cypin and MAP2 immunoreactivity were colocalized, indicating that DH neurons express cypin (Fig. 10). Interestingly, in the LDH, cypin-positive neurons were primarily found in the superficial layers of the SC. Only occasional cypin-positive neurons were present in the inner layers of the dorsal horn. Quantification of the fluorescent signal showed that injury or B9 treatment does not alter cypin immunoreactivity (Fig. 10). These data indicate that B9 treatment does not reduce neuropathic pain by altering cypin protein expression.

## Discussion

Our results demonstrate that transient treatment with the cypin inhibitor, B9, during acute/subacute phases after SCI has long-lasting effects and alleviates mechanical pain sensitivity in injured mice. Cypin inhibition has no impact on locomotor function, lesion volume, and astrocyte reactivity at the lesion site, suggesting that B9 treatment does not cause exacerbation of some of the other SCI outcomes.

Inhibition of cypin may act via multiple mechanisms to alleviate mechanical pain sensitivity. For example, inhibition of GDA activity results in decreased accumulation of the pain-associated molecule, UA, and buildup of the protective molecule, guanosine (Yuan et al., 1999; Paletzki, 2002; Akum et al., 2004; Fernandez et al., 2008). Furthermore, mechanical hyperalgesia occurs as a result of AMPA receptor and mGlu receptor coactivation and downstream signaling. In line with this, overexpression of cypin results in changes to AMPA receptor signaling (Rodriguez et al., 2021), and inhibition of cypin by B9 may act to block AMPA receptor signaling responsible for mediating mechanical hyperalgesia.

Glial activation also plays a role in mediating neuropathic pain. Activation of microglia and astrocytes that are present both proximal and distal to the lesion site is associated with the onset and maintenance of neuropathic pain (Shiao and Lee-Kubli, 2018). However, we did not detect glial activation in the LDH at 21 dpi since GFAP, and Iba-1 levels in SCI-sustaining mice were not significantly different than sham controls. The lack of glial activation in the LDH at 21 dpi agrees with earlier studies that indicate that in our SCI model, GFAP levels in the LDH are transiently increased at 8 dpi but subside to sham levels by 28 dpi (Pallottie et al., 2018). B9 treatment did not alter GFAP and Iba-1 levels in the LDH of injured or sham mice. However, it is possible that B9 treatment alters other glial responses relevant to the modulation of pain. This possibility requires further investigation. Of note, in contrast to the LDH, we found that GFAP levels are increased at the injury epicenter of the same mice. This finding is consistent with the well-known astrogliosis that occurs at the lesion site. However, B9 treatment does not alter GFAP levels at this site, suggesting that cypin inhibition does not impact astrocyte reactivity or astrogliosis.

The presence of cypin-immunoreactive neurons, particularly in the superficial layers, is of interest since A∂ afferents project to these layers and carry information from mechanical or mechanothermal nociceptors (Todd, 2010). We did not observe a change in cypin levels in these neurons in SCI-sustaining mice compared with shams, and B9 treatment did not have any effects on cypin-immunopositive neurons, at 21 dpi. It should be noted that B9 has been tested using both purified recombinant protein and neuronal extracts, and it significantly slows the initial rate and decreases the Vmax of guanine deamination by cypin; however, due to the fact that cypin is only expressed in a subset of neurons in the SC, it is technically difficult to measure the effect of B9 in SC tissue from mice. Additionally, mice express urate oxidase, and UA is rapidly converted to allantoin, making UA difficult to measure. We searched for allantoin measurement kits; however, none are commercially available. Based on data on B9 action on the recombinant protein and in neuronal extracts, modulation of cypin activity, but not expression, by B9 does not appear to be a mechanism underlying the antinociceptive effects of B9.

An important factor that must be considered when evaluating the results of this study is timing after injury. Aside from locomotor testing, all other analyses were performed at 21 dpi. However, it is likely that there are B9-mediated changes that occur at earlier stages post-SCI. Thus, it is possible that cypin inhibition induces transient molecular changes that are not detectable at 21 dpi and that treatment with B9 produces changes to signaling cascades that last beyond its immediate mechanism of action.

The present study focused on mechanical pain, as a first step, to determine whether cypin inhibition is a promising approach to treat SCI-induced neuropathic pain. The encouraging findings of the present investigations warrant future studies to determine whether B9 is effective in the treatment of other pain modalities following SCI. Investigations with larger animal cohorts and additional inhibitors are needed to determine whether cypin inhibition is a novel therapeutic avenue for the alleviation of SCI-induced neuropathic pain.

## Data Availability

The data presented in this study are available upon request from the corresponding author.

## Synthesis

Reviewing Editor: Deanna Smith, University of South Carolina Decisions are customarily a result of the Reviewing Editor and the peer reviewers coming together and discussing their recommendations until a consensus is reached. When revisions are invited, a fact-based synthesis statement explaining their decision and outlining what is needed to prepare a revision will be listed below. The following reviewer(s) agreed to reveal their identity: NONE.

The authors describe a potentially novel way to affect neuropathic pain, which might have a significant impact on many patients suffering from the debilitating condition after spinal cord injury. The experiments are well defined and the rationale is logical. The data are convincing and the interpretation of the results are appropriate. However, the reviewers and I agree that there are some issues that need to be addressed:

(i) The authors make the case that no interventions have been identified to combat spinal cord injury associated pain. This is not entirely accurate, as it has been well documented that activity-based interventions are effective in alleviating pain or even, as some experiments suggest, preventing mechanical allodynia from developing in the first place. Please include the appropriate references and discuss this intervention in the context of the data shown here

(ii) The authors describe in Figure 1 the cypin activator H9, but no experiments or data are shown that are related to this factor. Please remove H9 from Figure 1 or add experiments that are related to this activator.

(iii) The authors do not demonstrate that the transient treatment with the cypin inhibitor, B9 actually inhibits cypin. They merely refer to previous work where the function of B9 has been tested in a different context. A more thorough explanation and evaluation of the action of the Cypin inhibitor in the context of the in vivo application in SC injury is needed.

(iv) The manuscript only describes one specific kind of pain. This is not sufficient to make a generalized statement about Cypins action. Please acknowledge this limitation and explain why only one specific pain paradigm was chosen for this study.

(v) The authors discuss that cypin inhibition might affect dendrites but previous works showed that B9 has no effect on dendrites and did not affect dendrite length in vitro cultures. Please explain these seemingly contradicting arguments.
